# Computed tomography manifestations in super early stage 2019 novel coronavirus pneumonia

**DOI:** 10.1177/0284185120924806

**Published:** 2020-05-21

**Authors:** Shan Hu, Zhen Li, Xu Chen, Chang-Hong Liang

**Affiliations:** 1 The Second School of Clinical Medicine, Southern Medical University, Guangzhou, PR China; 2 Department of Radiology, Guangdong General Hospital, Guangdong Academy of Medical Sciences, Guangzhou, Guangdong, PR China; 3 Department of Radiology, General Hospital of the Yangtze River Shipping, Wuhan, PR China; 4 Department of Urology, The First Affiliated Hospital, Sun Yat-Sen University, Guangzhou, PR China

**Keywords:** 2019 novel coronavirus, thin chest CT, ground-glass opacity, reverse transcription polymerase chain reaction

## Abstract

**Background:**

The recent outbreak of pneumonia cases in Wuhan, PR China, was caused by a novel beta coronavirus, the 2019 novel coronavirus (COVID-19).

**Purpose:**

To summarize chest computed tomography (CT) manifestations of the early stage of COVID-19 infection and provide a piece of reliable imaging evidence for initial screening and diagnosis.

**Material and Methods:**

From 10 January 2020 to 10 February 2020, we continuously observed chest CT imaging of 14 patients with clinically suspected new coronavirus infection in the two weeks after onset of symptoms. Ground-glass opacity (GGO), consolidation, reticular pattern, and ground-glass mimic nodules in each patient’s chest CT image were recorded.

**Results:**

We enrolled 14 patients, of which nine patients had the infection confirmed by reverse transcription polymerase chain reaction (RT-PCR). Five patients were highly suspected of infection. All cases had epidemiological evidence. GGO was a dominant imaging manifestation in the initial days of infection. GGO performance accounts for 40% in 1– 2 days, 90% in 3– 6 days, and 85% in 7– 10 days. With disease progression, consolidation appeared on follow-up CT. Consolidation performance accounts for 0% in 1– 2 days, 40% in 3– 6 days, and 71% in 7– 10 days. The lesions are mostly near the pleura. The number of lesions and the extent of the lesions increased as the disease progressed.

**Conclusion:**

Patients with novel coronavirus pneumonia have characteristic CT features in the initial stage of infection, which can be used as an essential supplement for nucleic acid examination.

## Introduction

The recent outbreak of pneumonia cases in Wuhan, PR China was caused by a novel beta coronavirus, the 2019 novel coronavirus (COVID-19) (1). As of 30 January 2020, the World Health Organization designated this outbreak as a global health emergency. By 5 February 2020, 24,554 confirmed cases were reported globally; 24,363 confirmed cases were reported in PR China (2). The population is generally susceptible. The age of adult patients is 25– 89 years, and most of them are aged 40– 60 years. Most infected patients complained of fever, dry cough, and myalgia or fatigue. Some cases can progress to severe respiratory distress syndrome; some patients had headache, runny nose, diarrhea, and other symptoms. Laboratory tests showed that the total number of white blood cells in the peripheral blood was normal or decreased; the lymphocyte count decreased. Most patients had elevated C-reactive protein and erythrocyte sedimentation (3). New coronavirus reverse transcription polymerase chain reaction (RT-CPR) can be detected in throat swabs, sputum, lower respiratory tract secretions, blood, and stool samples (4). Based on current epidemiological investigations, the latency period for COVID-19-infected pneumonia is generally 3– 7 days, with a maximum of 14 days. According to the “Diagnosis and Treatment Protocol for COVID-19 (Sixth Revised Edition)” distributed by the National Health Commission, the primary source of infection is people infected with the new coronavirus infection. Asymptomatic infections can also be a source of infection (5).

The disease has a long incubation period and some patients only show mild symptoms or none at all. Undoubtedly, this increases the difficulty of screening suspected patients, which makes it hard to control the spread of the disease. In clinical observation, we indeed found that some patients only present mild symptoms (6), such as fatigue or low fever in the first few days. Early detection and timely isolation are an effective method of blocking transmission.

The aim of the present study was to let peers understand the imaging manifestations of the incubation period of the disease or early stage of the disease. Even if the RT-PCR is negative, the patient who had these manifestations on computed tomography (CT) should be highly suspected to be infectious and remind clinical close observation and isolation to prevent further spread of the virus.

## Material and Methods

### Patients

From 10 January 2020 to 10 February 2020, we enrolled hospitalized patients highly suspected and confirmed of COVID-19. Suspected cases were identified as having fever or any acute respiratory symptoms, and close contact with a confirmed or probable case of COVID-19 in the 14 days before the onset of illness or history of travel, or contact with people from Wuhan within two weeks, or worked at or attended a healthcare facility in the 14 days before the onset of symptoms where patients with hospital-associated COVID-19 infections have been reported. Confirmed cases were defined as a positive result of RT-PCR assay based on the WHO interim guidance (7). Suspected cases excluded patients without follow-up CT. According to the Guidelines for the Diagnosis and Treatment of Novel Coronavirus (COVID-19) Infection by the National Health Commission (Trial Version 5) (5), we excluded severe and critically ill patients based on clinical diagnostic guidelines; all cases were mild. In the end, we enrolled 14 patients (four women, ten men) highly suspected of having and confirmed with COVID-19: three cases were from Guangzhou (Guangdong Province) and 11 cases were from Wuhan (Hubei Province). All cases were proved and signed informed consent was obtained from the patients.

### CT scan

All patients were placed in the supine position and scanning was performed at end-inspiration. One unit (HiSpeed Advantage; GE Medical Systems, Milwaukee, Wisconsin, USA) was used in eight patients with the following parameters: section thickness = 1 mm; gap = 10 mm; scanning time per section = 1 or 2 s; 120 kV; and 240 mA. Another unit (Lightspeed plus; GE Medical Systems) was used in three patients with the following parameters: section thickness = 1.3 mm; gap = 10 mm; scanning time per section = 1 s; 120 kV; and 264 mA. A third unit (Philips Ingenuity; Philips, Netherlands) was used in three patients with the following parameters: section thickness = 1.0 mm; gap = 10 mm; scanning time per section = 1 s; 120 kV; and 269 mA. The images were photographed at lung (window width = 1000–  1600 HU; window level = – 600 HU) and mediastinal (window width = 350 HU; window level = 35– 40 HU) settings.

### Clinical data

We recorded complaints and carried out laboratory tests of the 14 patients when they were admitted. All patients denied a history of respiratory diseases. Laboratory tests in all patients showed normal or decreased white blood cell counts. We recorded the times of each patient’s CT scans and each scan time, including CT before admission and follow-up CT after admission. The time of onset of symptoms and the results of the nucleic acid test were recorded.

### Review of CT images

All CT images were reviewed by two radiologists (radiologist Hu Shan, radiologist Li Zhen) using a viewing console. Decisions were reached by consensus. Each segment of the lung was evaluated for opacity. The location of the lesion was defined as peripheral if it was in the outer one-third of the lung; otherwise, it was defined as central.

Ground-glass opacity (GGO) was defined as increased lung parenchymal attenuation that did not obscure the underlying vascular architecture. Consolidation was defined as opacification in which the underlying vasculature was obscured (8). Reticular pattern consisted of either coarse linear or curvilinear opacities or fine subpleural reticulation without substantial GGO. Each lesion was magnified and examined for intralobular, interlobular septal, or peribronchovascular interstitial thickening. Attention was also paid to the presence of nodules or vascular congestion and thickening, pleural effusion, and bronchial wall thickening. Any other observed abnormalities were noted.

## Results

The 14 hospitalized patients consisted of four female patients and ten male patients. Nine patients had infection confirmed by RT-PCR (64%), of which case 2 and 5 were negative for the first time and positive for the second time. Five patients who were highly suspected of infection have the follow-up CT scans and negative PCR results until the last CT scan. All cases had epidemiological evidence. Only two of the cases were asymptomatic.

Two cases (cases 3 and 4) had the first CT scan without any symptoms; they requested self-inspection after close contact with infected patients. The earliest CT scans is on the day of the onset of symptoms; the latest scan is 14 days after the onset of symptoms. The latest time of the first CT scan is six days after the onset of symptoms. Twelve cases had CT scans before and after admission. Only two cases (cases 13 and 14) had CT scans after admission (Table 1).

**Table 1.** Patient characteristics.*

Case no.	Gender/Age (years)	E	Symptoms	RT-PCR	CT scans (n)	Scan day after the onset of symptoms
1	M/38	+	Fever, fatigue	–	2	1, 5
2	M/44	+	Fever, fatigue	+	2	1, 4
3	M/61	+	No symptoms	+	1	Unknown
4	F/60	+	No symptoms	+	1	Unknown
5	M/36	+	Dry cough with dizziness, fever	+	4	1, 4, 7, 14
6	M/37	+	Cough 5 days, fever	+	2	2, 4
7	M/32	+	Cough 4 days, fever	+	3	1, 3, 7
8	M/29	+	Fever, fatigue	–	2	1, 7
9	F/42	+	Fever 2 days	–	3	2, 5, 8
10	M/65	+	Fever 1 day	–	2	2, 7
11	F/64	+	Dry cough 3 days	–	2	3, 8
12	M/35	+	Fever with sore throat 5 days	+	2	5, 10
13	F/51	+	Cough 2 days, fever	+	1	6
14	M/30	+	Fever 2 days	+	1	4

Cases 1– 12 had CT scans before and after admission. Cases 13 and 14 only had CT scans after admission. The symptoms were complaints of patients at admission.

CT, computed tomography; E, epidemiological evidence; RT-PCR, reverse transcription polymerase chain reaction.

**Fig. 1.**
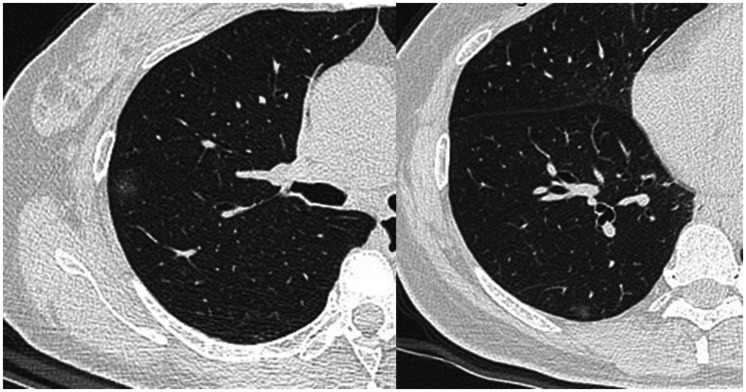
Pure ground-glass opacifications in case 4. The lesions are near the pleura.

**Fig. 2.**
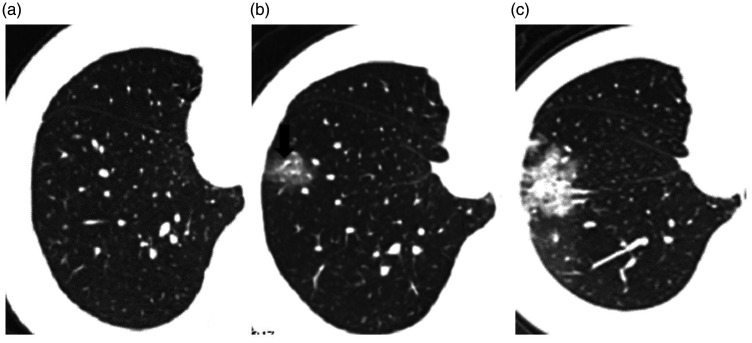
(a) CT scan two days after onset of symptoms; CT is negative. (b) CT scan five days after onset of symptoms, showing GGO with vascular congestion (arrow). (c) CT scan eight days after onset of symptoms, showing GGO with vascular congestion and part consolidation. CT, computed tomography; GGO, ground-glass opacity.

In the very early stage of the disease (1– 2 days after onset of symptoms), unilateral or bilateral localized inflammatory infiltration of the lung was most characterized by segmental or subsegmental GGOs (40%), which could be accompanied by vascular congestion, or interlobular septal thickening and interstitial changes. The lesions are mostly near the pleura (Fig. 1). However, no consolidation pattern was found 1– 2 days after the onset of symptoms. The CT scans for 5 (50%) cases were negative in the early stage and positive on follow-up CT, in which two cases showed a positive RT-PCR (Fig. 2). Two cases (20%) with asymptomatic infections were positive for CT and positive for RT-PCR (Table 2).

**Table 2.** Pattern of thin chest CT on days 1– 2 after onset of symptoms.

Case no.	RT-PCR	Symptoms	GGO	VC	Reticular pattern	Consolidation	Nodules	Location	
1	–	+	–	–	–	–	–	/	
2	–	+	–	–	–	–	+	P > C*	
3	+	–	+	+	+	–	–	C > P^†^	
4	+	–	+	–	–	–	–	P	
5	/	+	–	–	–	–	–	/	
6	/	+	–	–	–	–	–	/	
7	/	+	+	–	+	–	–	P	
8	/	+	–	–	–	–	–	/	
9	/	+	–	–	–	–	–	/
10	/	+	+	+	–	–	–	P	
Total	2 (2/10)	8 (8/10)	4 (4/10)	2 (2/10)	2 (2/10)	0	1 (1/10)		

C, central; CT, computed tomography; GGO, ground-glass opacification; P, peripheral; RT-PCR, reverse transcription polymerase chain reaction; VC, vascular congestion.

*More peripheral lesions than central lesions.

^†^More central lesions than peripheral lesions.

**Fig. 3.**
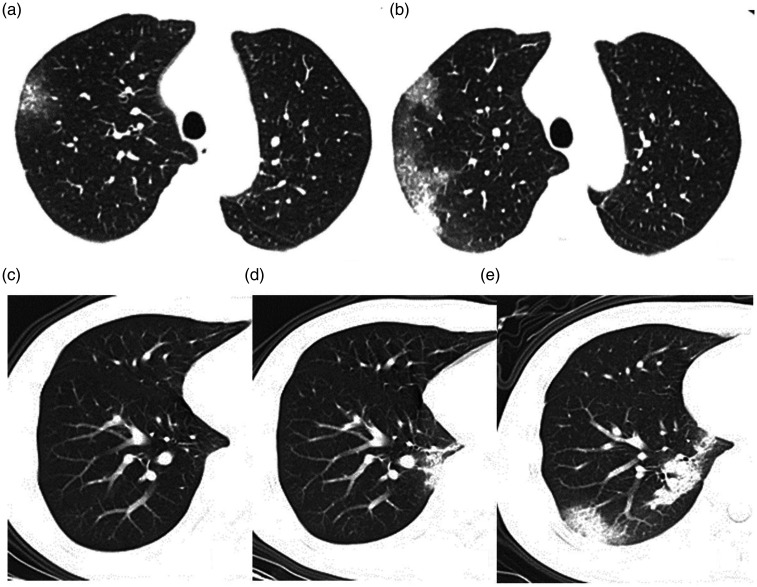
(a, b) Case 10. (a) CT scan two days after onset of symptoms (vascular congestion). (b) CT scan seven days after the onset of symptoms (part consolidation). (c– e) Case 7. (c) CT scan on the day of onset of symptoms (negative on this CT slice), (d) CT scan three days after onset of symptoms (GGO with a reticular parttern), (e) CT scan seven days after onset of symptoms (consolidation). Both cases show the number of lesions, and the extent of the lesions increased as the disease progressed. CT, computed tomography; GGO, ground-glass opacity.

In the progressive stage (3– 6 days up to 14 days), we found that the consolidation pattern appeared (40% in 3– 6 days, 71% in 7– 10 days), and the GGOs appeared in more cases gradually (90% in 3– 6 days, 85% in 7– 10 days). Eight cases showed GGOs with consolidation. The number of lesions and the extent of the lesions increased as the disease progressed. Imaging changes in COVID-19 pneumonia are rapid (at least two days) (Tables 3– 5 and Fig 3).

**Table 3.** Pattern of thin chest CT on days 3– 6 after onset of symptoms.

Case no.	RT-PCR	Symptoms	GGO	VC	RP	Consolidation	Nodules	Location
1	–	+	+	–	–	+	–	P
2	+	+	+	–	–	+	+	P > C*
12	+	+	+	–	–	–	–	P > C*
5	–	+	–	–	–	–	–	/
6	+	+	+	+	–	–	–	P
7	/	+	+	–	+	–	–	P
9	/	+	+	+	–	–	–	P
11	/	+	+	+	–	–	–	P
13	+	+	+	+	+	+	–	P
14	+	+	+	–	–	+	–	P
Total	5 (5/10)	10 (10/10)	9 (9/10)	4 (4/10)	2 (2/10)	4 (4/10)	1 (1/10)	

C, central; CT, computed tomography; GGO, ground-glass opacification; P, peripheral; RT-PCR, reverse transcription polymerase chain reaction; VC, vascular congestion.

*More peripheral lesions than central lesions.

**Table 4.** Pattern of thin chest CT on days 7– 10 after onset of symptoms.

Case no.	RT-PCR	Symptoms	GGO	VC	RP	Consolidation	Nodules	Location
12	+	+	+	–	–	+	–	P > C*
5	+	+	+	–	–	–	–	P
7	+	+	–	–	+	+	–	P
8	–	+	+	–	–	–	–	P
9	–	+	+	+	–	+	–	P
10	–	+	+	+	–	+	–	P
11	–	+	+	+	+	+	–	P
Total	3 (3/7)	7 (7/7)	6 (6/7)	3 (3/7)	2 (2/7)	5 (5/7)	0	

C, central; CT, computed tomography; GGO, ground-glass opacification; P, peripheral; RT-PCR, reverse transcription polymerase chain reaction;VC, vascular congestion.

*More peripheral lesions than central lesions.

**Table 5.** Pattern of thin chest CT on day 14 after onset of symptoms.

Case no.	RT-PCR	Symptoms	GGO	VC	RP	Consolidation	Nodules	Location
5	+	+	+	–	–	+	+	P > C*

C, central; CT, computed tomography; GGO, ground-glass opacification; P, peripheral; RT-PCR, reverse transcription polymerase chain reaction;VC, vascular congestion.

*More peripheral lesions than central lesions.

**Fig. 4.**
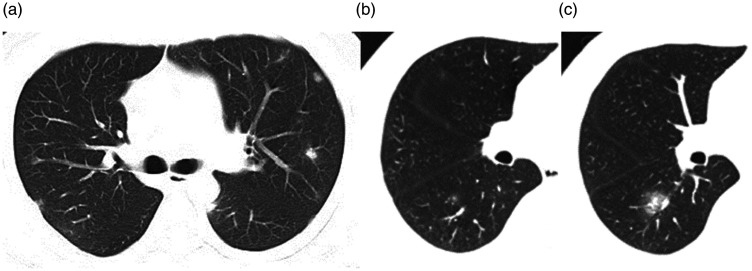
(a) Case 5 14 days after onset of symptoms. GGO mimics nodules in the bilateral lung. (b, c) Case 2. (b) CT scan one the day of onset of symptoms, (c) CT scan four days after onset of symptoms. A GGO mimicking a nodule is seen in the dorsal segment of the right lower lung in (b). The lesion progresses in (c).

GGOs mimicking nodules with ill-defined borders and GGOs were found in only a few cases (case 2 in 1– 2 days, case 5 in 14 days) (Fig. 4). No patients had pleural effusion(s), lymphadenopathy, or fibrosis and bronchial wall thickening in the initial phase of the disease.

RT-PCR results had no correlation with any CT findings (Table 6).

**Table 6.** The relationship between CT findings and the results of RT-PCR.

Case no.	RT-PCR	GGO	VC	RP	Consolidation	Nodules
1	–	– /+	– /–	– /–	– /+	– /–
2	+	– /+	– /–	– /–	– /+	+/+
3	+	+	+	+	–	–
4	+	+	–	–	–	–
5	+	– /– /+/+	– /– /– /–	– /– /– /–	– /– /– /+	– /– /– /+
6	+	– /+	– /+	– /–	– /–	– /–
7	+	+/+/–	– /– /–	+/+/+	– /+	– /– /–
8	–	– /+	– /–	– /–	– /–	– /–
9	–	– /+/+	– /+/+	– /– /–	– /-/+	– /-/–
10	–	+/+	+/+	– /–	– /+	– /–
11	–	+/+	+/+	– /+	– /+	– /–
12	+	+/+	– /–	– /–	– /+	– /–
13	+	+	+	+	+	–
14	+	+	–	–	+	–

CT, computed tomography; GGO, ground-glass opacification; RT-PCR, reverse transcription polymerase chain reaction; VC, vascular congestion.

## Discussion

To our knowledge, this is the first description focusing on thin chest CT manifestations of patients who are highly suspected of and confirmed with COVID-19 pneumonia in very early-stage disease with short-term review CT.

COVID-19 has different coronavirus-specific nucleic acid sequences from known human coronavirus species, which are shared with the severe acute respiratory syndrome (SARS)/Middle East respiratory syndrome (MERS) coronaviruses, a common ancestor that resembles the bat coronavirus HKU9-1. COVID-19 poses a significant public health risk for human transmission via the S protein– ACE2 (angiotensin-converting enzyme 2) binding pathway (9). In 2002– 2003, a SARS coronavirus-related epidemic occurred. ACE2 is also a potential SARS virus receptor and is a negative regulator of the renin-angiotensin system that affects vascular permeability. ACE2 is expressed in the lungs and kidneys and induces direct lung injury by involving ACE (10,11). Therefore, we infer that COVID-19 causes direct lung injury by involving ACE, which contributes to diffuse alveolar damage.

The CT signs of pulmonary viral infection will depend on the underlying pathologic process. According to the postmortem results in six fatal cases of SARS, macrophages featured strongly as the main neutrophil infiltrates in the alveoli and interstitium of these fatal cases, even in those with early disease, which suggests that proinflammatory cytokines released by macrophages may underlie the pathogenesis of SARS (12). Our results showed that GGO was the most important manifestation in the early stages of infection. With time, consolidation appeared on CT. This imaging progression may be consistent with the pathological mechanism of disease progression. Therefore, we assumed that the GGO was caused by the partial filling of air spaces, interstitial thickening, partial collapse of alveoli, normal expiration, or increased capillary blood volume. As transudate, exudate, or tissue replacing alveolar air, consolidation appeared on 3–14 days after the onset of symptoms. In addition, the lesions involved in subpleural are more common in the initial phase of the illness same as SARS (13), which may be related to the pathological mechanisms such as bronchioles and respiratory bronchioles surrounding the lung parenchyma that were affected in the early stages of viral pneumonia. Pleural effusion(s), lymphadenopathy, or fibrosis and bronchial wall thickening are not common findings in the coronavirus pneumonia (11). This is consistent with our findings.

The CT patterns of viral pneumonia are related to the pathogenesis of pulmonary viral infection. The influenza virus is an important human respiratory tract infection in the community. Influenza virus diffusely invades the respiratory epithelium, resulting in necrotizing bronchitis and diffuse alveolar damage, which manifests as poorly defined patchy or nodular areas of consolidation on CT. Respiratory syncytial virus (RSV) replicates in the nasopharyngeal epithelium, spreads to the lungs, and induces bronchiolitis with sloughing of epithelial cells of the small airways, which manifest as an airway centric distribution, with areas of tree-in-bud opacity and bronchial wall thickening, with or without consolidation along with the bronchovascular bundles (11). A typical CT scan of COVID-19 findings included pulmonary parenchymal GGO with consolidations. Pleural effusions, bronchial wall thickening, or lymphadenopathy are not common findings.

There are many papers about COVID-19 pneumonia in CT findings. The GGOs with consolidations are main findings, which is similar to these papers. In our research, GGOs mimic nodules with ill-defined border and ground-glass shadows around then nodules was found in the early stages. This performance has not been mentioned in other papers on CT manifestations of COVID-19 pneumonia. We are the first to discover this CT manifestation (14,15).

The status of RT-PCR in the diagnosis of new coronavirus pneumonia is unquestionable. In the present study, five cases were negative of RT-PCR; two cases were negative for the first time and positive for the second time. The current detection method uses fluorescent PCR technology to detect the content of new coronavirus nucleic acids in a patient’s biological sample. If the nucleic acid content exceeds a threshold (i.e. is positive), the patient can be considered infected with the new coronavirus. However, RT-PCR below a certain threshold (i.e. negative) cannot rule out novel coronavirus infections. The nucleic acid test result is affected by conditions such as sample quality, test timing, and level of technology. False-negative results are prone to occur as well as missed diagnoses. In our false-negative RT-PCR of first-time patients, it was possible that they were all in the early stages of the disease. The virus copy number in the sample is lower than the detection limit in the initial phase of the infection. Five cases had a false-negative and a positive CT scan with the progression of the next CT images; we infer that it was related to the quality of the sample or test timing. In addition, nucleic acid testing also has the disadvantages of high testing qualifications and long testing time.

In this epidemic, chest CT examinations played a pivotal role. Due to the high rate of missed diagnoses of plain chest X-ray, chest CT shows that the focus of early infection is sensitive; therefore, it is the primary method of screening and auxiliary diagnosis. Combining RT-PCR kits and chest CT examinations can significantly improve diagnostic sensitivity and specificity. In our cases, we found two cases without symptoms but positive on both CT and RT-PCR. Therefore, we infer that CT manifestations are earlier than clinical symptoms in a few patients with the COVID-19 infection. Thin chest CT examination has an irreplaceable role in preclinical screening.

Nevertheless, the CT scan cannot be used as a substitute for nucleic acid tests; we had two cases that were negative on CT scan on days 1– 2 of the initial phase, but were confirmed by RT-PCR and positive on follow-up CT. In addition, we found three patients with the imaging progression on CT with negative RT-PCR on days 1– 2. Thus, for patients with negatives on both CT and RT-PCR in the initial days of infection, it is suggested that these patients may need to continue being observed and isolated.

Although thin chest CT cannot be used as a substitute for nucleic acid detection to diagnose COVID-19 pneumonia, in epidemic areas where RT-PCR kits are limited, a large number of suspected cases cannot be diagnosed. The CT examination can be used as a robust supplementary measure, which is used to detect suspected infection in patients, and it helps to find more suspected cases for isolation as soon as possible.

It should be noted, however, that our patients probably represent those at the initial stage of the disease spectrum, primarily hospitalized patients, who have also developed appropriate indications or complications to justify serial evaluations with thin-section CT. Hence, natural selection bias may have been introduced into the study design. Other limitations include a limited number of cases in our study, and further studies can increase the sample size. Non-uniform scanning intervals among all patients caused by the retrospective nature of this study is another limitation of the study. However, because COVID-19 is highly infectious, it would have been inappropriate and against institutional infection control policy to systematically evaluate all COVID-19 patients.

In conclusion, our paper summarized CT abnormalities findings and temporal pattern changes of initial phases in COVID-19. GGO is mainly a manifestation of thin chest CT in the initial stage of COVID-19. Imaging changes in COVID-19 pneumonia are rapid, with the virus replicating massively and the inflammatory response getting worse. The number of lesions and the extent of the lesions on the image gradually increase, and consolidation appears on the CT image. Therefore, patients with COVID-19 pneumonia have characteristic CT features in the initial stage of infection, which can be used as an essential supplement for nucleic acid examination.
